# GlnR Dominates Rifamycin Biosynthesis by Activating the *rif* Cluster Genes Transcription Both Directly and Indirectly in *Amycolatopsis mediterranei*

**DOI:** 10.3389/fmicb.2020.00319

**Published:** 2020-03-03

**Authors:** Xinqiang Liu, Yuanyuan Liu, Chao Lei, Guoping Zhao, Jin Wang

**Affiliations:** ^1^CAS Key Laboratory of Synthetic Biology, Institute of Plant Physiology and Ecology, Shanghai Institutes for Biological Sciences, Chinese Academy of Sciences, Shanghai, China; ^2^University of Chinese Academy of Sciences, Beijing, China; ^3^Shanghai Tolo Biotechnology Company Limited, Shanghai, China; ^4^Department of Microbiology and Li Ka Shing Institute of Health Sciences, The Chinese University of Hong Kong, Shatin, Hong Kong; ^5^College of Life Sciences, Shanghai Normal University, Shanghai, China

**Keywords:** *Amycolatopsis mediterranei*, rifamycin biosynthesis, GlnR, nitrate-stimulating effect, *rifZ*, *rifK*

## Abstract

Because of the remarkable efficacy in treating *mycobacterial* infections, rifamycin and its derivatives are still first-line antimycobacterial drugs. It has been intensely studied to increase rifamycin yield from *Amycolatopsis mediterranei*, and nitrate is found to provide a stable and remarkable stimulating effect on the rifamycin production, a phenomenon known as “nitrate-stimulating effect (NSE)”. Although the NSE has been widely used for the industrial production of rifamycin, its detailed molecular mechanism remains ill-defined. And our previous study has established that the global nitrogen regulator GlnR may participate in the NSE, but the underlying mechanism is still enigmatic. Here, we demonstrate that GlnR directly controls rifamycin biosynthesis in *A. mediterranei* and thus plays an essential role in the NSE. Firstly, GlnR specifically binds to the upstream region of *rifZ*, which leads us to uncover that *rifZ* has its own promoter. As RifZ is a pathway-specific activator for the whole *rif* cluster, GlnR indirectly upregulates the whole *rif* cluster transcription by directly activating the *rifZ* expression. Secondly, GlnR specifically binds to the upstream region of *rifK*, which is also characterized to have its own promoter. It is well-known that RifK is a 3-amino-5-hydroxybenzoic acid (AHBA, the starter unit of rifamycin) synthase, thus GlnR can promote the supply of the rifamycin precursor by directly activating the *rifK* transcription. Notably, GlnR and RifZ independently activate the *rifK* transcription through binding to different sites in *rifK* promoter region, which suggests that the cells have a sophisticated regulatory mechanism to control the AHBA biosynthesis. Collectively, this study reveals that GlnR activates the *rif* cluster transcription in both direct (for *rifZ* and *rifK*) and indirect (for the whole *rif* cluster) manners, which well interprets the phenomenon that the NSE doesn’t occur in the *glnR* null mutant. Furthermore, this study deepens our understanding about the molecular mechanism of the NSE.

## Introduction

Being a broad-spectrum antibiotic with unique property of inhibiting the prokaryotic RNA polymerase activity (Wehrli et al., 1968; Campbell et al., 2001), rifamycin and its derivatives (e.g., rifampicin, rifabutin, rifapentine, and rifaximin) are still broadly used as the first-line antimycobacterial agents (Rothstein, 2016). To increase rifamycin yield, multiple strategies have been tested, including both engineering the producer *Amycolatopsis mediterranei* (a rare actinomycete) (Peano et al., 2014; Kumari et al., 2016) and optimizing fermentation conditions (Jiao et al., 1979; Lee et al., 1983; Mejia et al., 1998). Among those tested conditions, supplementation of nitrate was found to be able to remarkably stimulate rifamycin production, which has become known as the “nitrate-stimulating effect (NSE)” (Jiao et al., 1979). This phenomenon was first reported by our laboratory about 40 years ago, and has been widely exploited in the industrial production of rifamycin SV.

With the assistance of stable isotope tracing technology, it was found that the nitrogen atom of nitrate can be incorporated into the rifamycin at last, and both intracellular nitrogen metabolites and the biosynthesis of rifamycin precursors are greatly promoted upon nitrate supplementation (Jiao et al., 1984; Jin and Jiao, 1986). Later, to better understand rifamycin biosynthesis, the rifamycin biosynthetic gene cluster (*rif* cluster) and the complete genome of *A. mediterranei* were sequenced (August et al., 1998; Zhao et al., 2010). The *rif* cluster, consisting of 43 genes (i.e., from *rifS* to *rifZ*, Supplementary Figure S1) (Floss and Yu, 1999; Li et al., 2017), includes genes involved in the synthesis of the starter unit AHBA (3-amino-5-hydroxybenzoic acid), assembly and modification of the polyketide backbone, downstream processing and conversion of rifamycin, and resistance (Yu et al., 1999; Xu et al., 2003, 2005; Floss and Yu, 2005; Floss et al., 2011; Yuan et al., 2011). In addition, the genome annotation results of *A. mediterranei* U32 indicate that the *rif* cluster contains two canonical transcriptional regulators: the LuxR-family transcriptional regulator RifZ and the TetR-family transcriptional regulator RifQ (Zhao et al., 2010). Recently, our studies have revealed that RifZ is a pathway-specific regulator of the *rif* cluster and directly activates the transcription of all *rif* cluster genes (Li et al., 2017), and RifQ could reduce the intracellular toxicity of rifamycin via directly regulating the expression of the rifamycin exporter-encoding gene *rifP* (Lei et al., 2018).

About the molecular mechanism of NSE, our previously published results of the RNA-seq analysis in *A. mediterranei* U32 have provided more comprehensive insights: supplementation of nitrate is able to remarkably increase the transcripts of the *rif* cluster genes to accumulate more rifamycin-biosynthesis enzymes; furthermore, the transcriptions of some other genes (such as *glnA* and *nas* operon) are also profoundly activated by nitrate supplementation to furnish more precursors for rifamycin biosynthesis (Shao Z.H. et al., 2015). Accordingly, in that work we speculate that nitrate could stimulate the rifamycin production via increasing both the precursor supply and the biosynthetic enzyme accumulation (Shao Z.H. et al., 2015). In addition, our previous genetic analyses have established that GlnR may participate in the nitrate-mediated regulation on rifamycin biosynthesis, as the deletion of *glnR* from *A. mediterranei* resulted in the loss of NSE (Yu et al., 2007); however, the underlying molecular mechanism remains ill-defined.

In actinomycetes, GlnR is an OmpR-type transcriptional regulator, and those early studies show that most GlnR-regulated genes (e.g., *amtB*, *nirB*, *nasA*, *ureA*, *glnA*, and *ald*) are involved in the assimilation and utilization of a variety of nitrogen sources, such as ammonium, nitrate and urea (Fink et al., 2002; Tiffert et al., 2008; Wang and Zhao, 2009; Wang et al., 2012, 2013, 2014). Accordingly, the GlnR in actinomycetes is mainly considered as a global nitrogen metabolism regulator. Later, increasing evidences demonstrate that GlnR also regulates the transcription of other genes involved in carbon metabolism, antibiotic biosynthesis and so on (Liao et al., 2015; Qu et al., 2015; Shao Z. et al., 2015; Cen et al., 2016; He et al., 2016; You et al., 2017; Liu et al., 2019).

In this study, the *in vivo* analyses clearly reveal that GlnR positively regulates *rif* cluster transcription and rifamycin biosynthesis in *A. mediterranei* U32. Then the EMSAs and DNase I footprinting assays show that GlnR can specifically bind to the upstream regions of genes *rifK* and *rifZ*, respectively, which lead us to disclose that both genes in fact have their own promoters on the basis of primer extension assays. It is well-known that RifK is responsible for the rifamycin starter unit AHBA biosynthesis, and RifZ is the pathway-specific regulator for the whole *rif* cluster transcription. Therefore, one may easily conclude that GlnR both directly and indirectly regulates *rif* cluster genes transcription to control rifamycin biosynthesis, which well interprets our previous findings that the deletion of *glnR* resulted in the loss of NSE in *A. mediterranei*.

## Results

### GlnR Positively Regulates Rifamycin Biosynthesis Through Activating the Transcription of *rif* Cluster

To explore the role of GlnR in the NSE in *A. mediterranei* U32, we carefully compared the time course of bacterial growth and rifamycin yield between the *glnR*^–^ and *glnR*^+^ strains. The *glnR* null mutant (Δ*glnR*) was constructed using double crossover recombination (Yu et al., 2007; Lin et al., 2014). To obtain the *glnR* complemented strain (*glnR*+), the *glnR* gene (with its native promoter) was integrated into the chromosome of Δ*glnR*, employing the integrative plasmid pDZL803 as the vector (Lin et al., 2014; Li et al., 2017). As a control, Δ*glnR* was integrated with the vector to obtain Δ*glnR*/803 (Lin et al., 2014), which was used to investigate the influence of the empty plasmid on both bacterial growth and rifamycin production.

**FIGURE 1:**
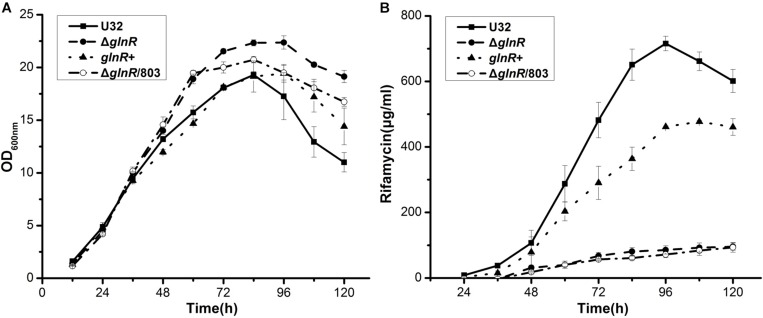
Effects of the *glnR* deletion on bacterial growth and rifamycin production in *A. mediterranei* when cultured in liquid Bennet medium containing 80 mM KNO_3_. **(A)** Growth curves of four *A. mediterranei* strains were determined by measuring the OD_600_ values of the cultures. **(B)** The rifamycin yield was measured with the differential spectrophotometric method. Error bars represented the SD from three independent biological replicates. U32, the wild type; Δ*glnR*, the *glnR* null mutant; *glnR*+, Δ*glnR* complemented with an intact *glnR*; Δ*glnR*/803, *glnR* null mutant integrated with the vector pDZL803.

In liquid Bennet medium supplemented with 80 mM nitrate, the above four strains showed similar growth rates before 48 h (Figure 1A). After 48 h, the wild type U32 and *glnR*+ strains started to produce a large amount of rifamycin, but Δ*glnR* and Δ*glnR*/803, both of which showed a higher growth rate, produced significantly less rifamycin (only ∼15% of the wild type) by 120 h (Figure 1). Moreover, although the Δ*glnR* showed a little higher growth rate than the wild type U32 when grown in liquid Bennet medium without nitrate, it had a significantly lower yield of rifamycin than the yield of U32 (Supplementary Figure S2), which was similar with the above results under nitrate condition. Based on these results, one may conclude that GlnR has weakly negative effect on bacterial growth but positively regulates the rifamycin biosynthesis in *A. mediterranei*.

**FIGURE 2:**
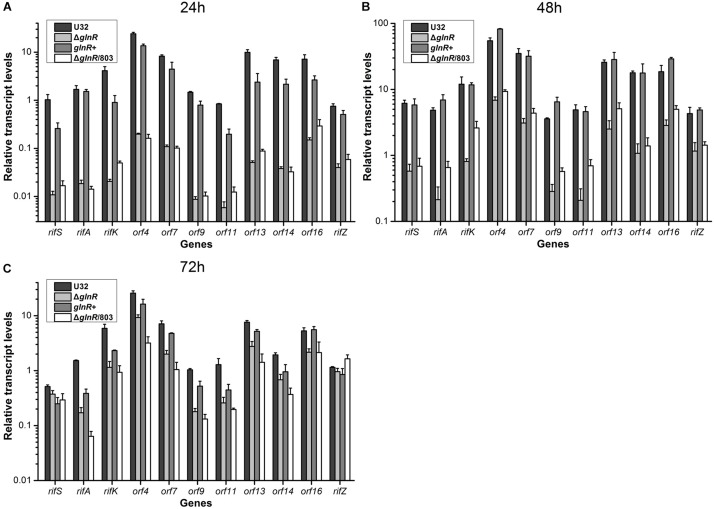
Transcriptional analyses of the *rif* cluster genes in *A. mediterranei* strains when cultured in liquid Bennet medium containing 80 mM KNO_3_. The relative transcriptional levels of 11 representative genes in the *rif* cluster were measured using qRT-PCR at 24 h **(A)**, 48 h **(B)**, and 72 h **(C)**, respectively. The *rpoB* gene was used as the internal control, and the transcriptional level of the *rifS* gene in U32 at 24 h was used as the reference (i.e., its value was adjusted to 1). Error bars represented the SD from at least three independent biological replicates. U32, the wild type; Δ*glnR*, the *glnR* null mutant; *glnR*+, Δ*glnR* complemented with an intact *glnR*; Δ*glnR*/803, Δ*glnR* integrated with the empty plasmid pDZL803.

Then we used quantitative real-time PCR (qRT-PCR) to determine the potential regulatory influence of GlnR on the transcription of *rif* cluster. The same four strains were cultured in liquid Bennet medium supplemented with 80 mM nitrate, and samples were taken for RNA extraction at 24, 48, and 72 h, which respectively represented the early mid-logarithmic phase, mid-logarithmic phase, and early stationary phase (ref to the Figure 1A). Our previous work has established that the *rif* cluster consists of 10 polycistronic operons (Supplementary Figure S1) (Li et al., 2017), and here we chose at least one gene from each of these operons to perform transcription analysis. It was found that the transcriptional levels of nearly all of the tested genes in *glnR*^–^ strains (i.e., Δ*glnR* and Δ*glnR*/803) were remarkably reduced compared to those in the wild type, especially at 24 h, when their ratio was only ∼1% (Figure 2). When the mutant Δ*glnR* was complemented with an intact *glnR*, the transcriptional levels of most *rif* genes in *glnR*+ were restored to levels comparable with the wild type (Figure 2). In addition, the transcriptional levels of *rif* genes were compared between the wild type U32 and Δ*glnR* when grown in liquid Bennet medium without nitrate supplementation, and it was found that the tested genes in the Δ*glnR* again showed profoundly reduced transcriptional levels at both 24 h and 48 h (Supplementary Figure S3). These results clearly demonstrate that GlnR is a transcriptional activator for the whole *rif* cluster.

### GlnR Indirectly Activates the Whole *rif* Cluster Transcription Through Regulating the Expression of the Pathway-Specific Regulator RifZ

**FIGURE 3:**
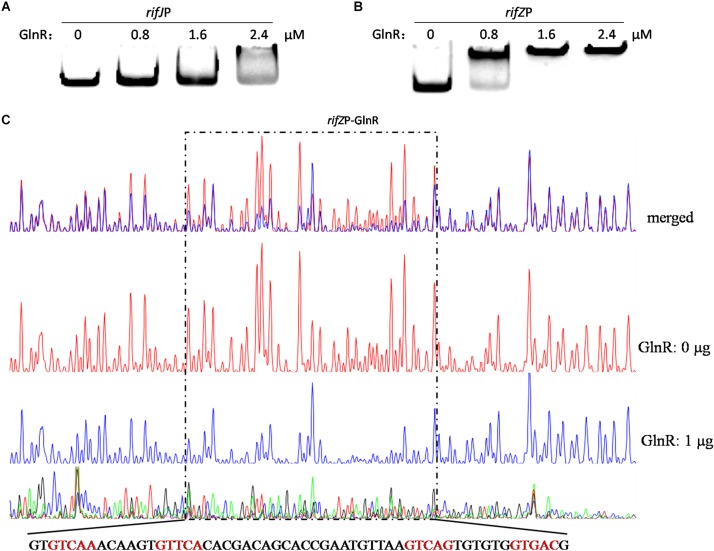
Analyses of the GlnR binding affinity to the *rifZ* promoter region. **(A)** Analysis of the GlnR binding affinity to the promoter region of the *rifJ-rifZ* operon using EMSA. **(B)** Analysis of the GlnR binding affinity to the *rifZ* promoter region using EMSA. **(C)** Characterization of the GlnR-protected sequences in the *rifZ* promoter via DNase I footprinting assay. Reactions were performed either without (the control reaction, red line) or with (the experimental reaction, blue line) the addition of the GlnR protein, and the electrophoretograms were merged to ease the identification of the GlnR-protected region, which was indicated by a dashed box. The precise DNA sequence was shown at the bottom, and the four predicted typical GlnR-binding sites were highlighted in red.

Given our findings that GlnR regulates the transcription of all the *rif* genes we tested, and considering the known fact that RifZ is a pathway-specific activator for the whole *rif* cluster, we wondered whether GlnR may indirectly regulate the transcription of all *rif* genes by activating the transcription of the *rifZ* gene. Note that the *rifZ* co-transcribes with the upstream gene *rifJ* and these two genes together form an operon (Li et al., 2017), we then performed EMSA and DNase I footprinting assay to test whether GlnR directly bound to the promoter region of this operon but both obtained negative results (Figure 3A and Supplementary Figure S4), which indicated that GlnR cannot directly bind to the promoter of the *rifJ*-*rifZ* operon.

Noticeably, the length of the intergenic region between *rifJ* and *rifZ* is more than 600 bps. More importantly, from the previously published RNA-seq data set (Shao Z.H. et al., 2015), we found that the transcription of *rifZ* had a more than 10-fold increase relative to the transcription of *rifJ* (Supplementary Table S1). Thus, we speculate that in addition to sharing the promoter of the operon, *rifZ* probably also has its own promoter to be operated by transcriptional regulators. To test this hypothesis, we firstly performed EMSA and found GlnR was able to specifically bind to the DNA sequence immediately preceding the gene *rifZ* (i.e., the *rifZ* promoter) (Figure 3B). Subsequently, we employed the DNase I footprinting assay to precisely determine the GlnR-protected DNA sequence in the *rifZ* promoter, and identified a 56-bp region (Figure 3C). In this region, we found two DNA sequences (GTCAC-n6-CTGAC, TGAAC-n6-TTGAC), which well meet the criteria of the GlnR binding sites (i.e., the fourth base should be ‘A’) (Wang et al., 2015). Here both the EMSA and DNase I footprinting results indicate that GlnR can specifically bind to the *rifZ* promoter and thereby directly operate the transcription of the pathway-specific regulator-encoding gene *rifZ*.

### Transcription Initiation Site Analysis Reveals *rifZ* Has Its Own Promoter

**FIGURE 4:**
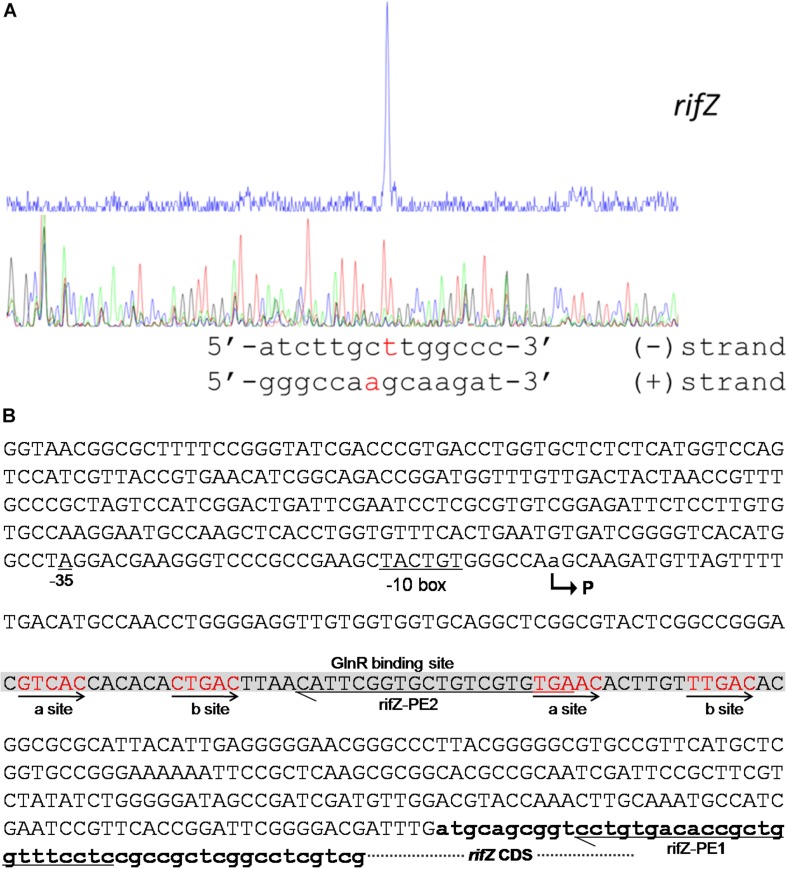
Characterization of the *rifZ* promoter. **(A)** Characterization of the *rifZ* TIS via primer extension assay. During the assays two different primers were used, and the primer extension result using the primer rifZ-PE2 was shown here. Locations of both primers as well as the primer extension result using the primer rifZ-PE1 could be found in Supplementary Figure S5. After the primer extension reaction, the samples were analyzed by the electrophoresis, whose results were shown in the upper panel. Sequencing results of the promoter region were shown in the bottom panel, and the identified TIS of *rifZ* was marked in red. **(B)** Schematic of the *cis*-elements in the *rifZ* promoter. The TIS of *rifZ* was indicated by a bent arrow and labeled as “P”. The GlnR-protected sequences were shown in gray background and the GlnR-binding sites (a site and b site) were shown in red and indicated by arrows. Those two primers (rifZ-PE1 and rifZ-PE2) used for primer extension assays in Supplementary Figure S5 were also labeled.

To fully understand this GlnR-mediated transcriptional regulation mechanism, we characterized the transcription initiation site (TIS) of the gene *rifZ* using the primer extension assay. In detail, two specific primers were designed to be complementary to either the *rifZ* coding DNA sequence (CDS) or the *rifZ* promoter region (Figure 4B and Supplementary Figure S5A), and both the primer extension results showed that the transcription of *rifZ* began from an adenine (A) site, which was located at the −328th nucleotide (nt) position relative to the translational start site of *rifZ* (Figure 4 and Supplementary Figures S5B,C). In addition, we also verified the *rifZ* TIS via qRT-PCR using two pairs of primers located upstream and downstream of the TIS, respectively. And results showed that the transcriptional level of the TIS downstream region was much higher than that of the upstream region (Supplementary Figure S5D), which further proves this TIS characterized by the primer extension assay.

Therefore, the primer extension results further demonstrate that the gene *rifZ* undoubtedly has its own promoter. Although the DNase I footprinting-confirmed GlnR binding sequences are positioned downstream of the *rifZ* TIS, we have already established that GlnR positively regulates the transcription of *rifZ* on the basis of the genetic experimental results above (Figures 1, 2). Therefore, we speculate that here the GlnR-mediated regulation is unlikely to accord with a typical activation model, wherein an activator binds to the sequence preceding a gene’s TIS and recruits the RNA polymerase complex to initiate the transcription.

### GlnR Directly Activates the Transcription of the AHBA Synthase-Encoding Gene *rifK*

Besides *rifZ*, we also tested whether GlnR bound to the promoter regions of other genes in the *rif* cluster. Firstly, our previous work has established that the *rif* cluster consists of 10 polycistronic operons, and these operons in fact shares only six promoter regions as four pairs of them are divergently transcribed (refer to the Supplementary Figure S1) (Li et al., 2017). Then, we re-analyzed the transcription profiles of all the *rif* genes in the previously published RNA-seq data set (Shao Z.H. et al., 2015), and found a fascinating phenomenon in their transcription levels: besides the gene *rifZ* mentioned above, there are still six intra-operonic genes had a ≥2-fold transcription increase relative to the transcription of their immediately preceding gene (Supplementary Table S1). Thus, it appears that in addition to sharing the promoter of the given operon, these six genes probably also have their own promoter. Finally, coupled with another two experimentally verified intra-operonic promoters of *rifP* (Lei et al., 2018) and *rifZ* (just characterized in the Figure 4), there are collectively at least 14 promoters within the *rif* cluster.

**FIGURE 5:**
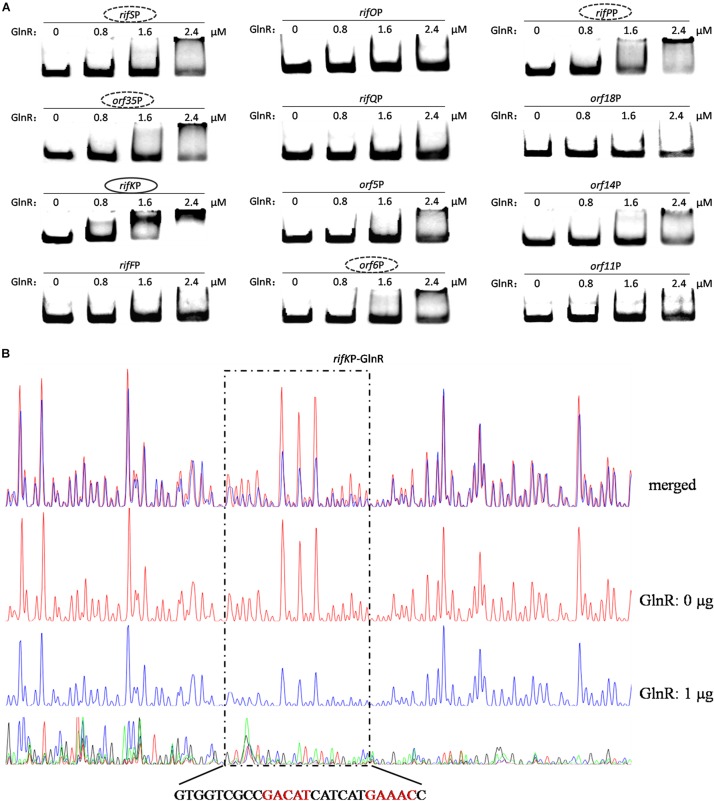
Analyses of the GlnR binding affinity to the *rifK* promoter region. **(A)** Analyses of the GlnR binding affinity to 12 promoter regions within the *rif* cluster using EMSA. To prevent the non-specific binding between the GlnR and probes, 2 μg sheared salmon sperm DNA was added into each reaction. Based on the amount of band shift, putative GlnR-targeted promoters were indicated by circles (with strong binding affinity) and dashed circles (with weak binding affinity). **(B)** Characterization of the GlnR-protected sequences in the *rifK* promoter via DNase I footprinting assay. The reactions were performed either without (the control reaction, red line) or with (the experimental reaction, blue line) the addition of the GlnR protein. The precise DNA sequences protected by GlnR were shown at the bottom, and the two predicted typical GlnR-binding sites were highlighted in red. Results of the DNase I footprinting assays of GlnR to other four putative targeted promoters (i.e., the dashed circles in Figure 5A) could be found in Supplementary Figure S6.

Since the two promoters of *rifZ* and *rifJ*-*rifZ* operon had been respectively tested above, here we only analyzed the GlnR binding affinity to the other 12 promoter regions using EMSA. The EMSA results showed that GlnR was able to significantly bind to the *rifK* promoter with a relatively higher binding affinity (Figure 5A). Furthermore, from the Figure 5A, it can be found that GlnR might also bind to another four promoters (*rifS*P, *orf35*P, *rifP*P and *orf6*P), but the binding affinity to them was much reduced relative to the promoters of both *rifK* and *rifZ*.

To verify these (possibly) positive EMSA results in Figure 5A and further understand the binding behavior, we next performed the DNase I footprinting assay to precisely determine the GlnR binding sequences in the five promoter regions, and found that GlnR only specifically protected a region in the *rifK* promoter (Figure 5B) but protected no region in the other four promoters (where the GlnR binding affinity was much reduced during the EMSAs) (Supplementary Figure S6). Therefore, these DNase I footprinting results lead us to accurately identify the true positive results of EMSAs, where too much regulatory protein easily generate false positive results.

**FIGURE 6:**
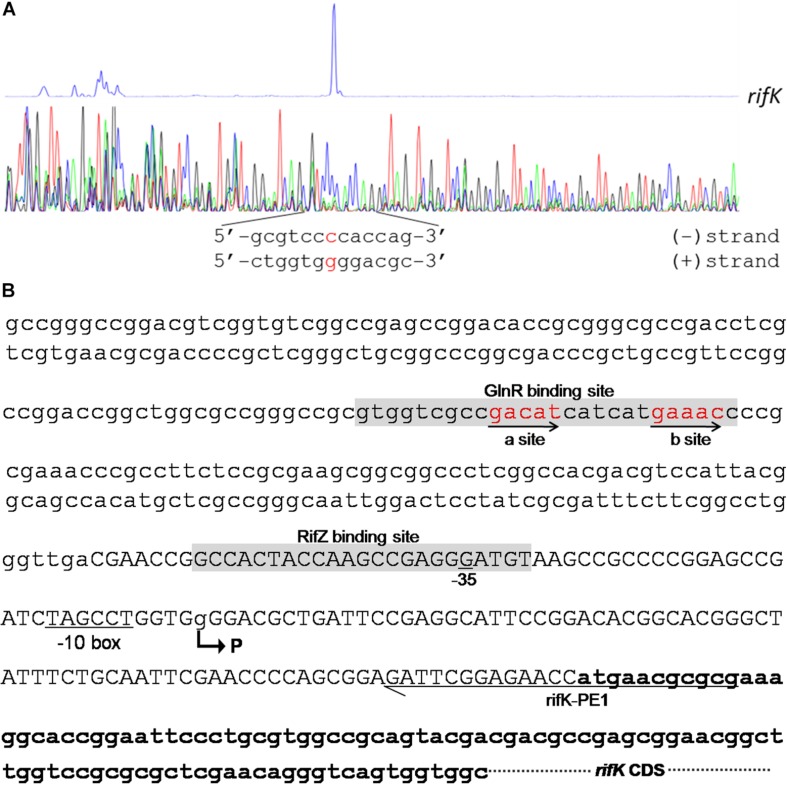
Characterization of the *rifK* promoter. **(A)** Characterization of the TIS of *rifK* via primer extension assay. **(B)** Schematic of the *cis*-elements in the *rifK* promoter. The TIS of *rifK* was indicated by a bent arrow and labeled as “P”. Both the GlnR-protected and RifZ-protected sequences were shown in gray background, and the typical GlnR-binding sites (a site and b site) were shown in red and indicated by arrows. The primer (rifK-PE1) used for the primer extension assay in Figure 6A was also labeled.

In the *rifK* promoter, GlnR protected a region from the −280th nt to the −255th nt relative to the *rifK* translational start site (Figure 6B), which did not overlap with the protected region by RifZ, another direct activator for *rifK* transcription (Li et al., 2017). And in the GlnR-protected region, a sequence of “GACAT-n6-GAAAC” was found, which well meets the criteria of the GlnR binding sites (Wang et al., 2015). As both GlnR and RifZ activate *rifK* transcription and their binding sequences are not overlapped, we speculate that these two regulators might be cooperative in regulating the transcription of *rifK*. Thereafter, to clarify the relative importance of GlnR and RifZ for the expression of *rifK*, we measured the transcriptional profiles of *rifK* in three strains (U32, Δ*glnR* and Δ*rifZ*) when cultured in liquid Bennet medium with 80 mM nitrate. Interestingly, it was found that the *rifK*’s transcriptional level in Δ*rifZ* was a little higher than in Δ*glnR* at 24 h, while its level in Δ*rifZ* was much lower than in Δ*glnR* at 48 and 72 h (Supplementary Figure S7). These results indicate that GlnR may be relatively more important for the *rifK* transcription comparing with RifZ during the early phase, and then the RifZ gradually surpasses the GlnR and plays a dominant role in the *rifK* transcription.

### Transcription Initiation Site Analysis Reveals *rifK* Has Its Own Promoter

To further investigate the molecular mechanism of GlnR-mediated activation of the *rifK* transcription, we performed primer extension assay to identify the TIS of the gene *rifK* using a primer complementary to the −13th nt to the 11th nt of the *rifK* CDS, and found *rifK* transcribed from a guanine (G) site which is located at the −79th nt position relative to the *rifK* translational start site (Figure 6). Noticeably, the -10 box (TAGCCT) of the *rifK* promoter was very similar to the previously characterized −10 box of promoters in *S. coelicolor* and *A. mediterranei* (Yu et al., 2006; Wang et al., 2013). In addition, as the GlnR-protected region is located upstream of the −35 box in the *rifK* promoter (Figure 6B), we speculate that GlnR might activate the *rifK* transcription through recruiting the RNA polymerase complex using a mode just similar to other typical transcriptional activators.

## Discussion

The NSE, i.e., the phenomenon that nitrate-stimulated remarkable increase of rifamycin biosynthesis in *A. mediterranei*, was initially demonstrated by our laboratory about 40 years ago (Jiao et al., 1979). Then it was noticed that the NSE did not occur in the *glnR* null mutant, however, the underlying molecular mechanism is still unknown (Yu et al., 2007). In the present study, we uncover the mechanism of GlnR controlling the rifamycin biosynthesis in *A. mediterranei* U32 via multifarious experiments both *in vivo* and *in vitro*.

The phenotypic analyses (Figure 1 and Supplementary Figure S2) showed that, unlike the previous work in *Streptomyces* (He et al., 2016), the cellular growth under our cultivation conditions was not impaired by *glnR* deletion at all, which ruled out the impact of the growth factor on gene transcription and on antibiotic production, thus making our conclusions from this study (especially the Figures 1, 2 and Supplementary Figures S2, S3) more reliable. The Supplementary Figure S2B showed that Δ*glnR* had a significantly lower yield of rifamycin comparing with the wild type U32 when grown in liquid Bennet medium without nitrate, whereas in our previous work, the rifamycin yield of Δ*glnR* was a little bit higher than that of U32 when grown on Bennet agar plates without nitrate supplementation (Yu et al., 2007). Here we speculated this difference might be caused by the cultivation conditions (i.e., liquid medium *versus* solid medium).

During the transcriptional assays, we noticed that the transcriptional levels of *rif* genes in the wild type U32 stayed high till 72 h under nitrate condition (Figure 2) but had dropped dramatically by the time point 48 h without nitrate supplementation (Supplementary Figure S3). These results were quite consistent with our previous RNA-seq results, where the nitrate supplementation well maintained the high-level transcription of the *rif* cluster (a key character/mechanism of NSE) (Shao Z.H. et al., 2015). And from the Figure 2, we found that the high-level transcription of the *rif* cluster under nitrate condition is severely impaired by the *glnR* deletion (especially at 24 and 48 h), which demonstrates that one reason for GlnR being necessary for NSE is its remarkable up-regulation on the *rif* cluster transcription. Interestingly, the Supplementary Figure S3 shows that GlnR can also up-regulate the *rif* cluster transcription under no nitrate condition (i.e., NSE cannot occur), indicating that NSE needs other factors besides the GlnR-mediated regulation on the *rif* cluster transcription. Furthermore, the magnitude of the differences in transcriptional levels between the *glnR*^–^ and *glnR*^+^ strains decreased significantly over time (Figure 2 and Supplementary Figure S3), indicating that the *rif* cluster (especially *rifZ* gene) may be controlled by other unknown mechanisms as well, which will be the focus of our future research.

All of the *in vivo* evidence in this study have clearly demonstrated that GlnR is an activator for the transcription of *rifZ* and *rif* cluster. However, the *in vitro* experimental results show that the GlnR binding regions are located downstream of the *rifZ* TIS, which is different from the binding locations of the typical transcriptional activators. In fact, some previous reports have shown that transcriptional regulators can have binding sites downstream of the TIS but still function as activators, e.g., Rns (Munson and Scott, 2000), MetR (Cowan et al., 1993), PhoP (Liu et al., 1998; Qi and Hulett, 1998), DnaA (Szalewska-Palasz et al., 1998), and FleQ (Baraquet and Harwood, 2015), with multiple distinct proposed regulatory mechanisms. Therefore, here we speculate that GlnR probably also activates *rifZ* transcription via an atypical activating model, which is worthy of detailed investigation in the future.

In addition, based on the results of both EMSA and DNase I footprinting assay (Figures 3, 5), we noticed that GlnR had lower binding affinity for the *rifK* promoter than for the *rifZ* promoter. For a regulator, an increased number of binding sites in its target sequences usually indicates higher binding affinity. Quite consistent with the different GlnR binding affinities against the two promoters, there are four typical GlnR-binding sites in the *rifZ* promoter, but only two typical GlnR-binding sites in the *rifK* promoter.

**FIGURE 7:**
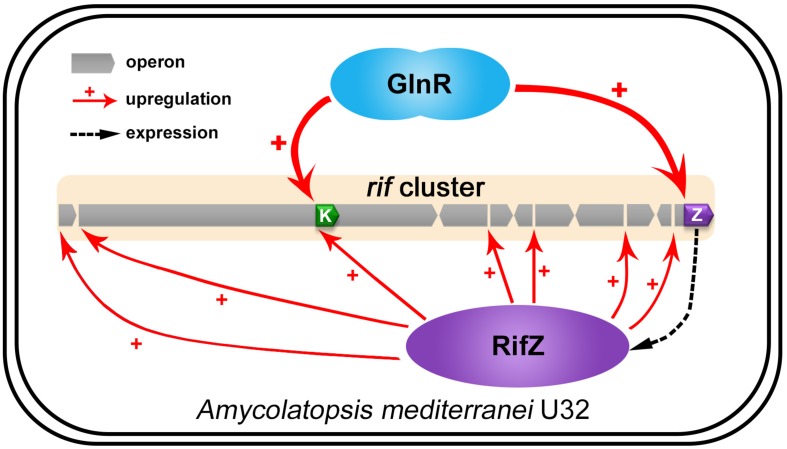
The model of GlnR-mediated activation for the *rif* cluster genes transcription in *A. mediterranei*. It has been established that RifZ functions as the pathway-specific activator for the whole *rif* cluster, therefore GlnR indirectly upregulates the transcription of all the *rif* cluster genes by activating the *rifZ* expression. Besides, the gene *rifK* encodes the AHBA synthase, GlnR therefore directly promotes the biosynthesis of the rifamycin starter unit AHBA by activating the *rifK* transcription. Taken together, GlnR both directly and indirectly activates the *rif* cluster genes transcription to enhance the rifamycin biosynthesis in cells, which further interprets the previous findings that the NSE did not occur in the *glnR* null mutant.

Here our study fully illustrates that the well-known global nitrogen regulator GlnR governs the rifamycin biosynthesis via activating the *rif* cluster transcription in both direct and indirect manners in *A. mediterranei* U32 (Figure 7). And combining the results of this study and some previous studies, nowadays we can have a more comprehensive insight that GlnR plays a significant role in NSE by acting as a global regulator to coordinate both primary nitrogen metabolism and secondary metabolism. In detail, when the U32 is cultured in Bennet medium with nitrate, which is a designated nitrogen-limited condition specially for U32 (Wang et al., 2014), GlnR activates the transcription of the primary nitrogen metabolism related genes (e.g., *glnA* and *nas* operon) to enhance the glutamine biosynthesis (Yu et al., 2006; Wang et al., 2013). And it also has been established that the glutamine provides the sole nitrogen atom for rifamycin biosynthesis (Jin and Jiao, 1986), therefore GlnR promotes the precursor supply for rifamycin biosynthesis by regulating the primary nitrogen metabolism. In addition to glutamine, the biosynthesis of AHBA is regulated in a very subtle way: on the one hand, GlnR directly activates the transcription of AHBA-synthase-encoding gene *rifK* via binding to its promoter; on the other hand, GlnR indirectly controls the *rifK* transcription *via* activating the expression of the pathway-specific regulator RifZ (Figure 7). As AHBA serves as the starter unit of rifamycin, such a subtle regulation on AHBA synthesis may further facilitate the rifamycin biosynthesis in cells. Besides the gene *rifK*, the GlnR also upregulates the whole *rif* cluster transcription indirectly via activating the *rifZ* expression (Figure 7). And the high-level transcription of the whole *rif* cluster could ensure the accumulation of rifamycin biosynthesis related enzymes in cells. In our opinion, it is quite compelling that the GlnR unexpectedly exerts such sophisticated regulation on rifamycin biosynthesis in *A. mediterranei*, which well interprets our previous findings that the NSE does not occur in the *glnR* null mutant.

In actinomycetes, GlnR was typically viewed as a global nitrogen metabolism regulator before, governing many nitrogen metabolism related processes (Fink et al., 2002; Tiffert et al., 2008; Wang and Zhao, 2009; Wang et al., 2012, 2013, 2014). Recently, several studies have reported that the GlnR homologs in *Streptomyces* regulate the biosynthesis of some antibiotics, e.g., actinorhodin, avermectin and validamycin (Qu et al., 2015; He et al., 2016). Here our study also shows that the GlnR in *A. mediterranei* directly controls the rifamycin biosynthesis. Therefore, taking all these findings together, one may easily conclude that GlnR can function as a global regulator instead of merely a nitrogen metabolism regulator in actinomycetes, coordinating both primary metabolism and secondary metabolism in cells.

## Materials and Methods

### Bacterial Strains, Media, and Primers

All the primers used in this study are listed in Table 1. And all the bacterial strains and plasmids used here are listed in Table 2. The *glnR* null mutant (Δ*glnR*) was obtained by double crossover recombination (Yu et al., 2007; Lin et al., 2014). The *glnR* complemented strain (*glnR*+) was constructed by integration of the *glnR* gene with its native promoter into Δ*glnR*, using the integrative plasmid pDZL803 (Li et al., 2017), and the construction procedure was described by Lin et al. (2014). *Escherichia coli* DH10B was used for construction of the recombinant plasmids, and was grown in Luria-Bertani (LB) medium (Sambrook and Russell, 2001). *A. mediterranei* strains were grown aerobically in Bennet medium (10 g/L glycerol, 10 g/L glucose, 2 g/L tryptone, 1 g/L yeast extract, 1 g/L beef extract, pH 7.0) at 30°C (Yang et al., 2001; Shao Z.H. et al., 2015). If necessary, antibiotics were added at the following concentrations (μg/ml): ampicillin, 100; and kanamycin, 50.

**TABLE 1:** Primers used in this study.

Name	Sequences (5′-3′)	
**Primers for qRT-PCR**		
qRT-rpoB-F	GAACCGCCCACCAAGGAGAGC	
qRT-rpoB-R	CGGTCAGCGTGCCGTTCTCG	
qRT-rifS-F	AGCAGCTGGTCGGTGAGGAT	
qRT-rifS-R	GGTTCTCCTTCTCCGCCTAC	
qRT-rifA-F	TCGAGGACATCCGCGACACT	
qRT-rifA-R	TGGTTGCGCAGGTTCCGGTA	
qRT-rifK-F	CCGGGCACCGAGGTCATC	
qRT-rifK-R	CCGGGTCGAGGTTGTAGG	
qRT-orf4-F	TCCGGTGGACCTGCTCAAGA	
qRT-orf4-R	AGTAGTCCTGGTCCTCGTGC	
qRT-orf7-F	GTGCTGGAACCGTGGATGTA	
qRT-orf7-R	CTGCAGGAAGTCGTACTGGT	
qRT-orf9-F	ACGCAACGGAATCCGCCATC	
qRT-orf9-R	CAGCGTCGAGGTCAGGATCT	
qRT-orf11-F	CGATGAACGCGATGGTGTCC	
qRT-orf11-R	AAGAAGAGCTACGCCGATCC	
qRT-orf13-F	GAAGAACCGTTGCCGCACAC	
qRT-orf13-R	ACCGTGAGCACGTCCTCGTA	
qRT-orf14-F	ACCGCGAACGACGTCATGGT	
qRT-orf14-R	TCGTCCACGTCGGCCTTGTA	
qRT-orf16-F	GTTCATGCAGGCGCTGGTCA	
qRT-orf16-R	GATGAGCAGCAGGTTGGCGA	
qRT-rifZ-F	ATGACCGCCGTCGAGCACAA	
qRT-rifZ-R	AGCGCCAGCACGACGTAACT	
qRT-rifZP1-F	GAGATTCTCCTTGTGTGCCA	
qRT-rifZP1-R	CACAGTAGCTTCGGCGGGAC	
qRT-rifZP2-F	CCTGGGGAGGTTGTGGTGGT	
qRT-rifZP2-R	AATGTAATGCGCGCCGTGTC	
**Primers for preparation of probes**		**Probe size (bps)**
rifSP-F	ACTCGAACGGCACCTCATCGTC	400
rifSP-R	CGCCGGTGGGAGCCATTACCAA	
orf35P-F	AGACCTGGAGGCGTTGATGCT	335
orf35P-R	CTCATCGCGGCCTCCACCTGT	
rifFP-F	CCCGTGCGGCGTCCACTGT	329
rifFP-R	CCACGTCGAACACGTTCACACCT	
rifKP2-F	CGGACGTCGGTGTCGGCC	433
rifKP-R	AGGGAATTCCGGTGCCTTTCG	
rifOP-F	ACGCGCTGGAAGGAAGCACCC	340
rifOP-R	AGAGTGCCCATGGCGCCGAAC	
rifPP2-F	CGGGGTGGCTCGCCGGATC	478
rifPP2-R	GACGAGGGCGTAGGCGTTGAT	
rifQP-F	CGCCGTGCTCAACTCGCGGTTC	344
rifQP-R	CCGTTTGCCCATCGTGGCCCTC	
orf5P-F	CGAGCTGGTTCGGGTTCATCATC	364
orf5P-R	TCATGGGGTCCTTCCTTTCGT	
orf6P-F	CGACGCCGTGGTCATCACCT	315
orf6P-R	ACCATGTCGTATCGATCCCTGC	
orf11P-F	CCAGCAGCGCCGAATAGCCCTC	345
orf11P-R	CTCCCAGTGGCCATAACGGCAAA	
orf18P-F	GGAGATGAAGAAGCTGCTG	223
orf18P-R	CCGCCATGGACATGACCGC	
orf14P-F	CGGCGACCACTTCCGGTGAGAAC	341
orf14P-R	CGGCTTCGTCACCAGTACCCCT	
rifJP-F	CGCCGACCAGCTCGCCCTC	391
rifJP-R	GCGTTTTCGGTCACTTTGGTCGT	
rifZP-F	GTCCCGCCGAAGCTACTGTG	362
rifZP-R	CCGCTGCATCAAATCGTCCC	
*Hin*cII-F	GTAAAACGACGGCCAGTGCG	
*Hin*cII-R	FAM-AACAGCTATGACCATGATTACGG	
**Primers for primer extension assay**		
rifK-PE1	FAM-CGCGCGTTCATGGTTCTCCGAATC	
rifZ-PE1	FAM-GAGGAAACCAGCGGTGTCACAGG	
rifZ-PE2	FAM-TCACACGACAGCACCGAATG	

**TABLE 2:** Strains and plasmids used in this study.

Strains or plasmids	Relevant characteristics	Sources
**Strains**		
*E. coli* DH10B	F^–^ *endA1 recA1 galE15 galK16 nupG rpsL* Δ*lacX74* Φ80*lacZ*ΔM15 *araD139* Δ(ara,leu)7697 *mcrA* Δ(*mrr-hsdRMS-mcrBC*) λ^–^	Lab stock
*E. coli* BL21(DE3)	F^–^ *ompT gal dcm lon hsdSB*(rB- mB-) λ(DE3)	NEB
*A. mediterranei* U32	Wild type; an industrial producer of rifamycin SV	Lab stock
*A. mediterranei*Δ*glnR*	*glnR* null mutant	Lin et al., 2014
*A. mediterranei glnR* +	Complementary strain; *glnR* null mutant complemented using an integrating vector with an intact *glnR*	Lin et al., 2014
*A. mediterranei*Δ*glnR*/803	*glnR* null mutant with empty vector	Lin et al., 2014
*A. mediterranei*Δ*rifZ*	*rifZ* null mutant	Li et al., 2017
**Plasmids**		
pEXAMR	GlnR expression plasmid with the *glnR* (*AMED_9008*) gene cloned into the *Eco*RI and *Hin*dIII sites of pET28a (+)	Wang et al., 2013
pDZL803	An integrative plasmid used in *A. mediterranei*, resistant to apramycin and hygromycin	Li et al., 2017
pUC18H	A plasmid derived from pUC18 with most restriction sites removed but the *Hin*cII site	Tolo Biotech.
pUC18H-rifSP	The promoter region of *rifS*-*rifT* operon cloned into the *Hin*cII site of pUC18H	This work
pUC18H-orf35P	The promoter region of *orf35*-*rifQ* operon cloned into the *Hin*cII site of pUC18H	This work
pUC18H-rifKP	The promoter region of the gene *rifK* cloned into the *Hin*cII site of pUC18H	This work
pUC18H-rifFP	The promoter region of the gene *rifF* cloned into the *Hin*cII site of pUC18H	This work
pUC18H-rifOP	The promoter region of the gene *rifO* cloned into the *Hin*cII site of pUC18H	This work
pUC18H-rifQP	The promoter region of the gene *rifQ* cloned into the *Hin*cII site of pUC18H	This work
pUC18H-orf5P	The promoter region of the gene *orf5* cloned into the *Hin*cII site of pUC18H	This work
pUC18H-orf6P	The promoter region shared by *orf6*-*orf3* operon and *orf7*-*orf8* operon cloned into the *Hin*cII site of pUC18H	This work
pUC18H-orf11P	The promoter region shared by *orf10*-*orf9* operon and *orf11*-*orf18* operon cloned into the *Hin*cII site of pUC18H	This work
pUC18H-orf18P	The promoter region of the gene *orf18* cloned into the *Hin*cII site of pUC18H	This work
pUC18H-orf14P	The promoter region shared by *orf13*-*orf19* operon and *orf14*-*orf15B* operon cloned into the *Hin*cII site of pUC18H	This work
pUC18H-rifJP	The promoter region shared by *orf16* and *rifJ*-*rifZ* operon cloned into the *Hin*cII site of pUC18H	This work
pUC18H-rifPP	The promoter region of the gene *rifP* cloned into the *Hin*cII site of pUC18H	This work
pUC18H-rifZP	The promoter region of the gene *rifZ* cloned into the *Hin*cII site of pUC18H	This work

### Measurement of the Growth Curve and Rifamycin Yield

After streak cultivation on Bennet plates for 4–5 days, *A. mediterranei* strains were transferred into 50 ml liquid Bennet medium containing ceramic beads (diameter: 2.5 mm) to avoid mycelia aggregation. After incubation in a shaker at 220 rpm for about 36 h, they were inoculated into 200 ml fresh liquid Bennet medium containing ceramic beads and 0/80 mM KNO_3_ with the final OD_600_ = 0.2. The growth curves of different strains were determined by measuring the OD_600_ values with NanoDrop 2000C (Thermo Fisher Scientific) every 12 h.

After the strains being inoculated into 200 ml liquid Bennet medium with 0 or 80 mM KNO_3_ for 24 h, 1 ml bacterial suspensions were taken from the fermentation broth at 12 h intervals. For determining the rifamycin yield, the samples were centrifuged and the supernatant was collected. Then the rifamycin yield was measured using the spectrophotometric method as previously described (Pasqualucci et al., 1970). Briefly, 20 μl supernatant was diluted in 180 μl acetate buffer containing 0.1% NaNO_2_ or 0.1% vitamin C, respectively. Then, the optical density was read at the wavelength of 447 nm, with the NaNO_2_ group taken as a blank control. Finally, the yield of rifamycin was calculated based on a standard curve prepared by using pure rifamycin SV as standards.

### RNA Extraction and Quantitative Real-Time PCR (qRT-PCR)

When *A. mediterranei* strains were grown in liquid Bennet medium with 0/80 mM KNO_3_ for 24, 48, and 72 h, 5–10 ml bacterial suspensions were collected and the mycelia were harvested at 4°C by centrifugation at 12,000 rpm for 5 min. Total RNA was then extracted from the mycelia using TRIzol reagent (Thermo Fisher Scientific) and SV Total RNA Isolation System (Promega), following the manufacturers’ instruction. Reverse transcription was performed with random primers using 1 μg total RNA as the template in a volume of 20 μl by the PrimeScript 1st Strand cDNA synthesis kit (TaKaRa), and the qRT-PCR was carried out with SYBR Premix Ex Taq II (Tli RNaseH Plus) (TaKaRa). At least three independent biological samples were tested in this experiment, and the relative transcriptional level of the tested genes was presented as means ± standard deviations (SD), using the *rpoB* gene as the internal control.

### Preparation of FAM-Labeled Probes

Promoter regions in *rif* cluster were PCR amplified with Phanta Max super-fidelity DNA polymerase (Vazyme), employing primers listed in Table 1. Then these PCR amplicons were separately cloned into the *Hin*cII site of pUC18H (Tolo Biotech.). After verification through DNA sequencing, these recombinant plasmids were used as the PCR templates to prepare FAM-labeled probes using primers of *Hin*cII-F and *Hin*cII-R (6-carboxyfluorescein [FAM] labeled). Finally, the FAM-labeled probes were purified by the Wizard SV gel and the PCR Clean-Up system (Promega), and quantified with NanoDrop 2000C (Thermo Fisher Scientific).

### Electrophoretic Mobility Shift Assay (EMSA) and DNase I Footprinting Assay

The heterogenous expression and purification of the His-tagged GlnR of *A. mediterranei* U32 were performed as previously described (Wang et al., 2013). EMSA was carried out using the purified His-tagged GlnR and FAM-labeled probes, following the same procedures as described in our previous work (Li et al., 2017). DNase I footprinting assay was performed by Tolo Biotech. according to the method described by Wang et al. (2012). In brief, each FAM-labeled probe of about 300 ng was incubated with different amounts of His-tagged GlnR for 30 min at 25°C. After DNase I digestion, the stop solution was added to stop the reaction. The promoter region was sequenced using specific primer with FAM labeled at the 5′-ends, following the procedures described before (Wang et al., 2012). The samples together with the sequencing products were then purified and loaded into an ABI 3130 sequencer for electrophoresis analysis. The electropherograms were analyzed with PeakScanner v1.0 software (Applied Biosystems) to characterize the precise DNA sequences protected by GlnR.

### Primer Extension Assay

Primer extension assay was carried out by Tolo Biotech (Lei et al., 2018). To prepare total RNA for primer extension assay, *A. mediterranei* U32 was inoculated into liquid Bennet medium with 80 mM KNO_3_ and incubated in a shaker at 30°C for about 36 h. Then, the mycelia were harvested by centrifugation, followed by extraction of the total RNA using a protocol the same as described above. For each assay, about 60 μg total RNA was used to characterize the TIS, employing FAM-labeled primers listed in Table 1. Sequencing analysis of the promoter region with the FAM-labeled primer was the same as that described before (Lei et al., 2018). The subsequent electrophoresis and analysis were the same as the procedures in the DNase I footprinting assay described above.

## Data Availability Statement

All datasets generated for this study are included in the article/Supplementary Material.

## Author Contributions

XL performed most of the experiments and prepared the draft. YL performed the primer extension assay. YL and XL performed the DNase I footprinting experiment. CL prepared the FAM-labeled probes. JW and GZ designed the study. JW revised the manuscript and supervised the whole project.

## Conflict of Interest

YL was employed by the company Shanghai Tolo Biotechnology Company Limited. The remaining authors declare that the research was conducted in the absence of any commercial or financial relationships that could be construed as a potential conflict of interest.

## Abbreviations

AHBA: 3-amino-5-hydroxybenzoic acid
CDS: coding DNA sequences
EMSA: electrophoretic mobility shift assay
NSE: nitrate-stimulating effect
qRT-PCR: quantitative real-time PCR
TIS: transcription initiation site.
