# Supplementary material for: GlnR Dominates Rifamycin Biosynthesis by Activating the rif Cluster Genes Transcription Both Directly and Indirectly in Amycolatopsis mediterranei
Source: Front Microbiol. 2020 Mar 3;11:319. doi: 10.3389/fmicb.2020.00319 (PMC7062684; doi:10.3389/fmicb.2020.00319)
Supplement: Supplementary file 1 [file Data_Sheet_1.PDF]

Supplementary Material

Table S1. The transcription-level fold change of a gene relative to its immediately preceding gene in the *rif* cluster[a]

Strand	Gene name	24 h[b]	48 h[b]
+	*rifS*	\	\
+	*rifT*	1.3	1.1
+	*orf35*	\	\
+	*orf0*	0.8	0.7
+	*rifA*	0.4	0.2
+	*rifB*	0.9	1.0
+	*rifC*	0.9	0.9
+	*rifD*	0.8	0.8
+	*rifE*	0.9	0.9
+	*rifF*	1.7	2.0
+	*orf1*	1.4	1.5
+	*rifG*	0.5	0.4
+	*rifH*	0.7	0.7
+	*rifI*	0.5	0.5
+	*rifK*	7.1	12.2
+	*rifL*	0.8	0.5
+	*rifM*	1.5	1.8
+	*rifN*	0.7	0.5
+	*rifO*	1.3	2.5
+	*orf2*	0.2	0.1
+	*rifP*	0.7	0.7
+	*rifQ*	2.4	1.8

-	*orf3*	0.2	0.2
-	*orf4*	0.9	0.9
-	*orf5*	5.8	4.1
-	*orf6*	\	\
+	*orf7*	\	\
+	*orf8*	0.8	0.6
-	*orf9*	1.7	1.9
-	*orf10*	\	\
+	*orf11*	\	\
+	*orf17*	0.8	0.6
+	*orf18*	2.9	4.1
-	*orf19*	0.5	0.5
-	*orf20*	0.7	0.6
-	*rifR*	0.9	0.7
-	*orf13*	\	\
+	*orf14*	\	\
+	*orf15A*	1.3	1.1
+	*orf15B*	0.8	0.7
-	*orf16*	\	\
+	*rifJ*	\	\
+	*rifZ*	11.9	24.4

[a] The transcriptional profiles of these 43 *rif* cluster genes are retrieved from the previous RNA-seq results published by our laboratory (Shao et al., 2015). Here we re-analyze the data and show the transcription-level fold change of a gene relative to its immediately preceding gene in this table. The fold changes ≥ 2 were marked in red, which indicates that the intra-operonic gene may have its own promoter (in addition to sharing the operon promoter).

[b] *A. mediterranei* U32 was cultured in liquid Bennet medium supplemented with 80 mM KNO_3. "\" represents the fold change is unavailable as it is the first gene in the operon.

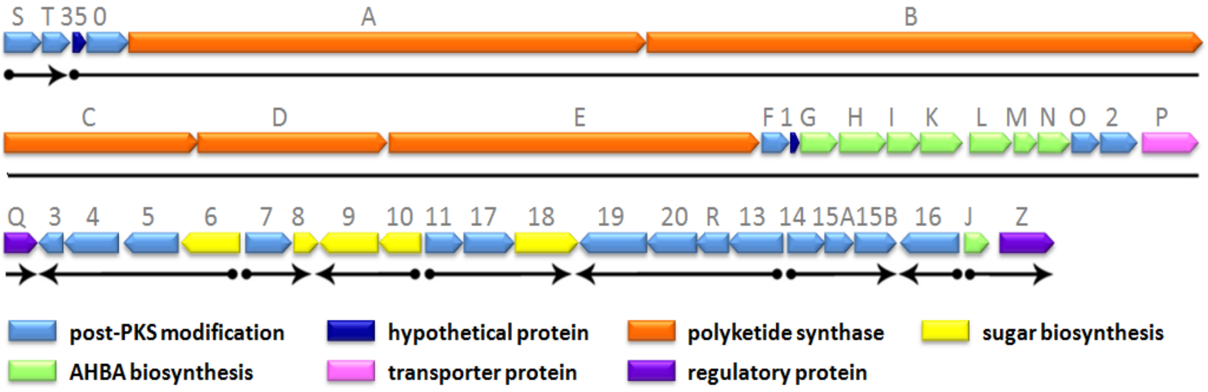

Figure S1. A schematic diagram of the *rif* cluster in *A. mediterranei* U32. Our previous co-transcription analyses results have established that the 43 genes of the *rif* cluster are divided into 10 operons (Li et al., 2017), and each operon is indicated by a black solid arrow, whose direction represents the transcriptional direction of each operon. Notably, the latter four pairs of operons are divergently transcribed, indicating each pair of them shares one promoter region.

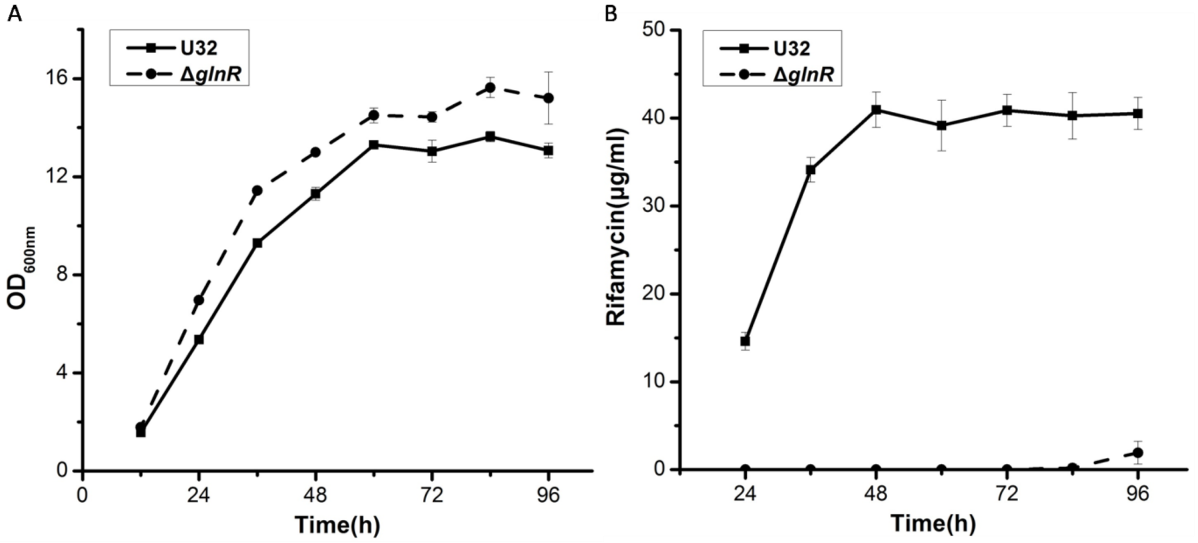

Figure S2. Bacterial growth and the rifamycin production of *A. mediterranei* strains grown in liquid Bennet medium without nitrate supplementation. (A) Growth curves of two *A. mediterranei* strains were determined by measuring the OD_{600} values of the cell cultures. (B) The rifamycin yield was measured using the spectrophotometric method as described in the Materials and Methods. U32, the wild type; Δ*glnR*, the *glnR* null mutant.

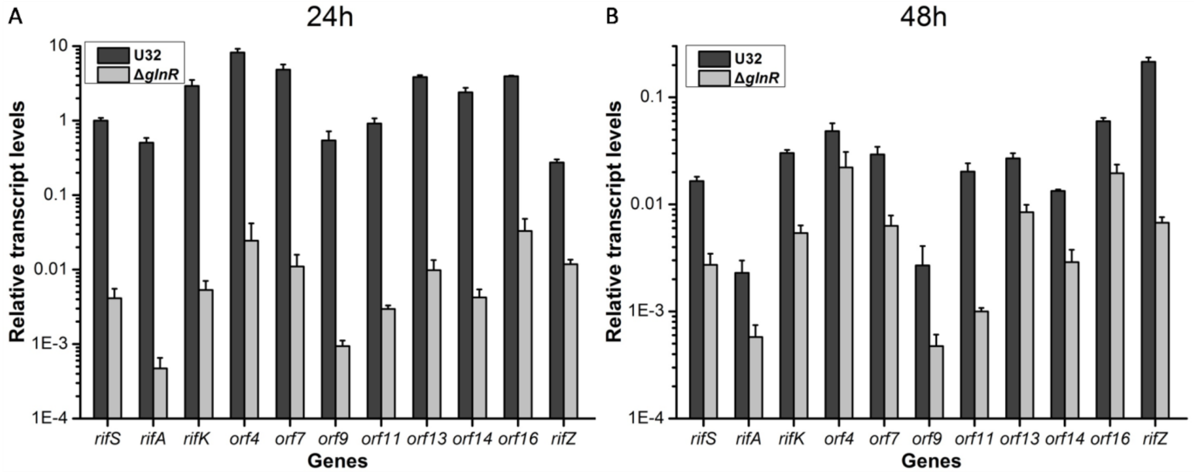

Figure S3. Transcriptional analyses of the *rif* cluster genes in *A. mediterranei* strains grown in liquid Bennet medium without nitrate supplementation. The relative transcriptional levels of 11 representative genes of the *rif* cluster were measured using qRT-PCR at 24 h (A) and 48 h (B). The *rpoB* gene was used as the internal control, and the transcriptional level of the *rifS* gene in U32 at 24 h was used as the reference (i.e., its value was adjusted to 1). U32, the wild type; Δ*glnR*, the *glnR* null mutant.

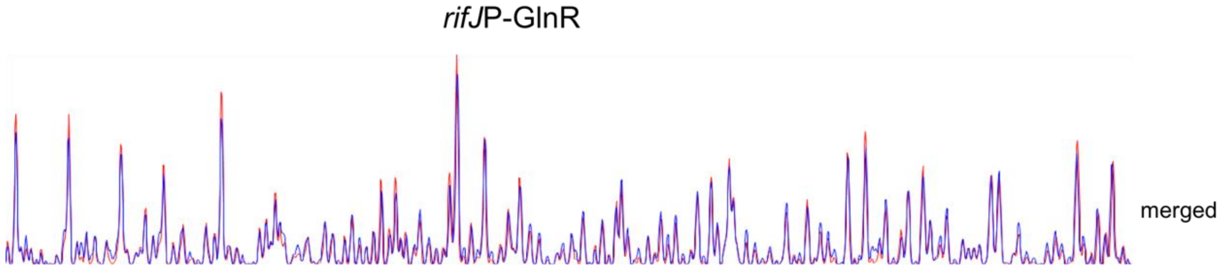

Figure S4. Analysis of the GlnR binding ability to the *rifJ-rifZ* operon promoter region by the DNase I footprinting assay. The electrophoretograms of both control reaction (without GlnR protein, red line) and experimental reaction (with 2 μg GlnR protein, blue line) were merged together. The GlnR-protected region could be identified in the distinct region between the two electrophoretograms (i.e., the intensity of the blue peaks was significantly lower than that of the red peaks). Here the merged electrophoretograms do not show obviously distinct region, indicating that GlnR does not specifically bind to the promoter region of *rifJ-rifZ* operon.

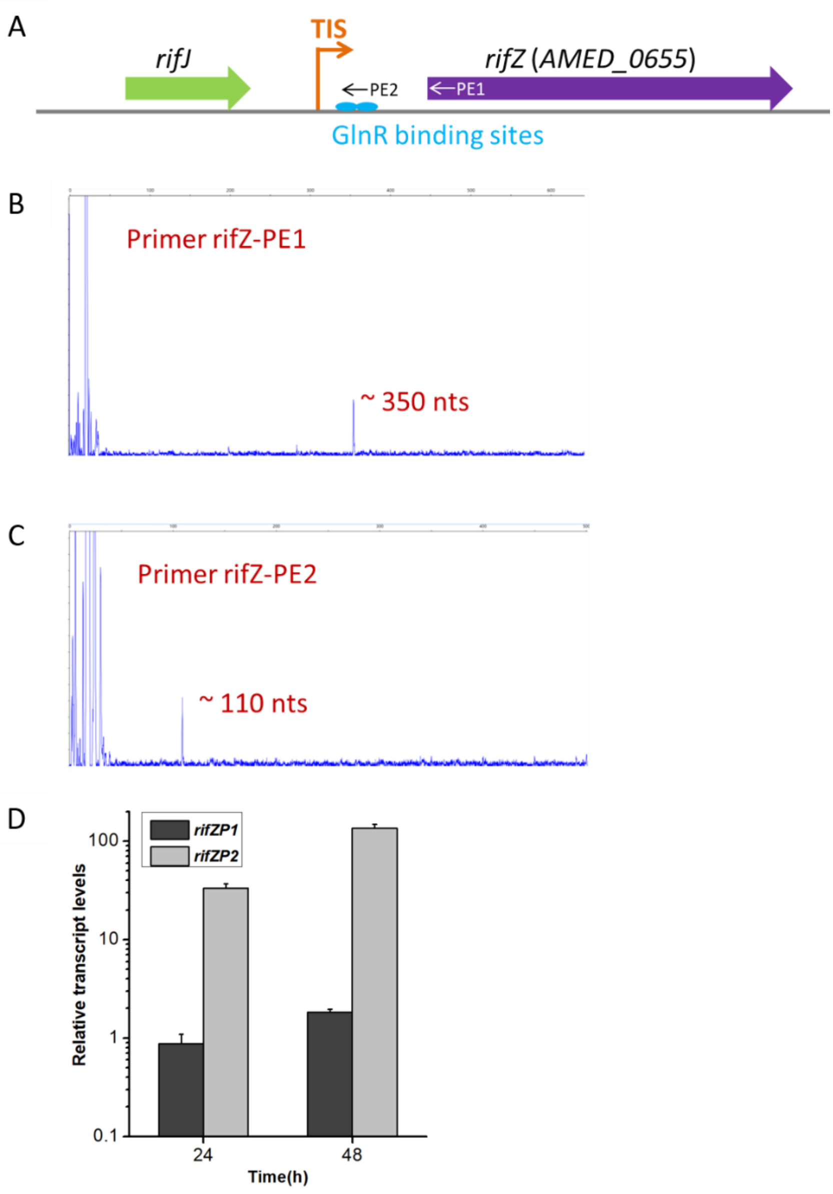

Figure S5. Characterization of the TIS of *rifZ*. (A) Schematic representation of the genes *rifJ* and *rifZ* as well as the promoter region of *rifZ*. The two primers used for primer extension assays, the GlnR-binding sites and the TIS of *rifZ* are respectively labeled in this schematic. (B and C) The results of primer extension assays using rifZ-PE1 and rifZ-PE2. Based on the size difference of the two reverse-transcribed products of these two primers, we conclude that the gene *rifZ* transcribes from the only site as labeled in Fig. S5A. (D) Verification of the *rifZ* TIS by qRT-PCR using the primers in Table 1. Here the total RNA used for analysis was from U32 grown in liquid Bennet medium with 80 mM nitrate. The *rifZP1* region is located upstream of the *rifZ* TIS, while the *rifZP2* region is located downstream of the *rifZ* TIS.

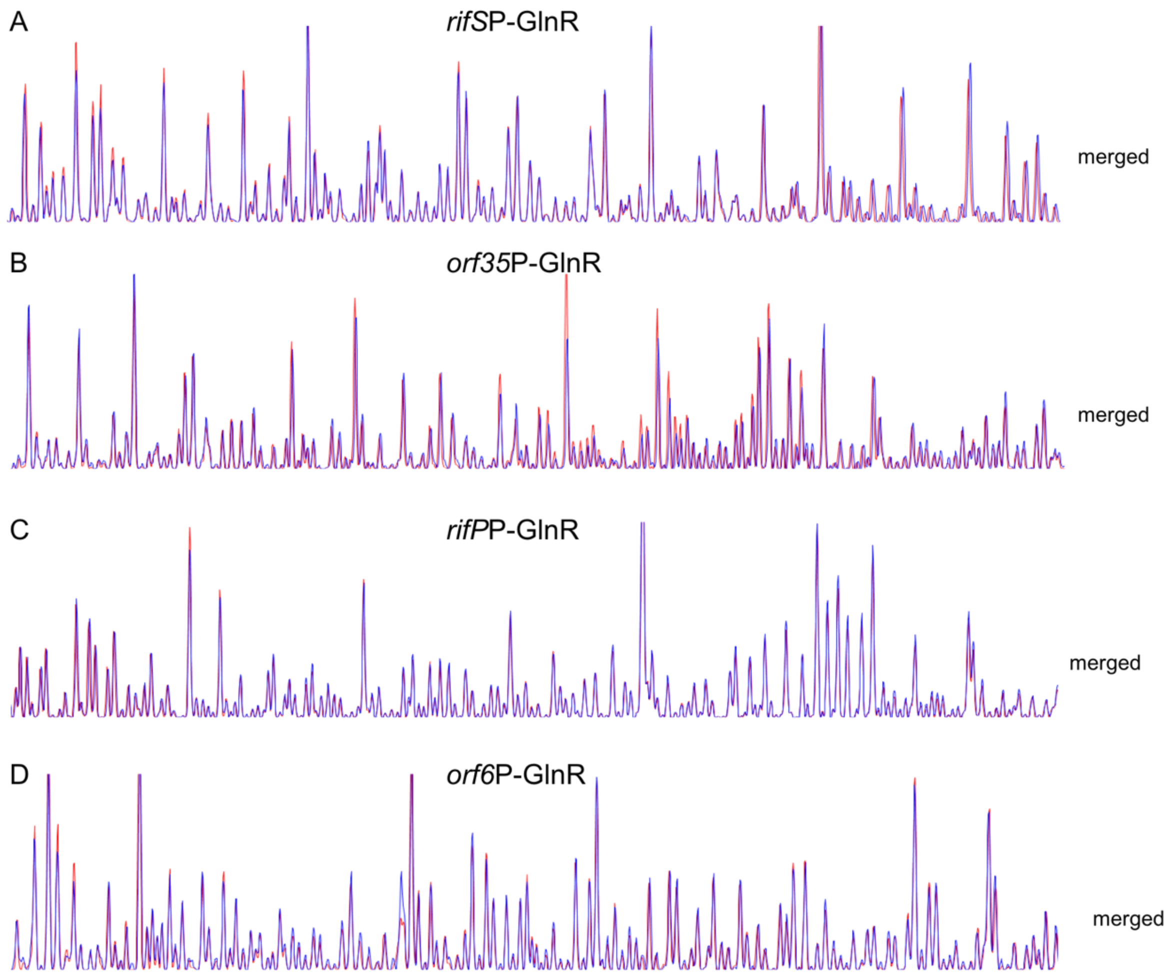

Figure S6. Analyses of the GlnR binding ability to its putative target promoter regions via DNase I footprinting assay. The results of GlnR to the four targets (i.e., *rifS*P, *orf35*P, *rifP*P and *orf6*P) are shown in A to D, respectively. During each DNase I footprinting assay, the electrophoretogram of the control reaction (without GlnR protein, red line) and the electrophoretogram of experimental reaction (with 2 μg GlnR protein, blue line) were merged together. Here the four merged electrophoretograms (A to D) show that none of them has obviously distinct regions between the two reaction conditions, indicating GlnR does not specifically bind to these four promoter regions.

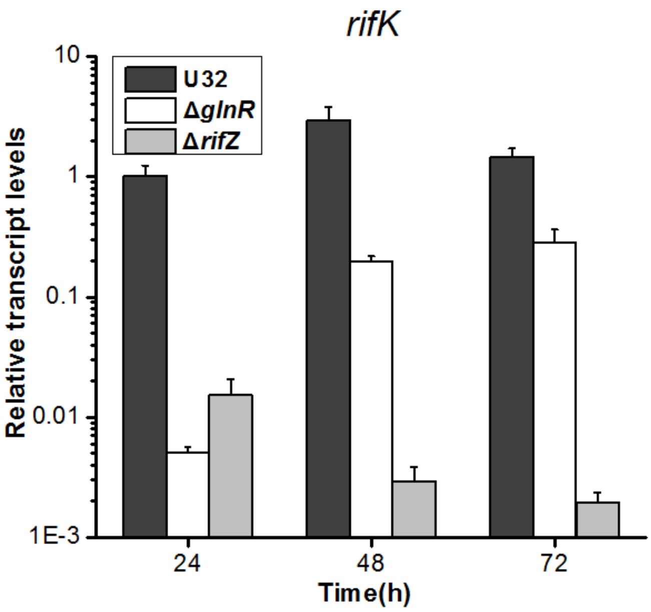

Figure S7. Transcriptional analyses of the gene *rifK* in U32, Δ*glnR* and Δ*rifZ* at 24 h, 48 h and 72h. Both of the Δ*glnR* and Δ*rifZ* were controlled by the wild type U32 and all of them were cultured in liquid Bennet medium containing 80 mM KNO_3. The *rpoB* gene was used as the internal control, and the transcriptional level of *rifK* in U32 at 24 h was used as the reference during analyses. U32, the wild type; Δ*glnR*, the *glnR* null mutant; Δ*rifZ*, the *rifZ* null mutant.

References

Li, C., Liu, X., Lei, C., Yan, H., Shao, Z., Wang, Y., et al. (2017). RifZ (AMED_0655) Is a Pathway-Specific Regulator for Rifamycin Biosynthesis in Amycolatopsis mediterranei. *Appl Environ Microbiol* 83(8). doi: 10.1128/AEM.03201-16.

Shao, Z.H., Ren, S.X., Liu, X.Q., Xu, J., Yan, H., Zhao, G.P., et al. (2015). A preliminary study of the mechanism of nitrate-stimulated remarkable increase of rifamycin production in Amycolatopsis mediterranei U32 by RNA-seq. *Microb Cell Fact* 14**,** 75. doi: 10.1186/s12934-015-0264-y.
